# Development the societal preference-based utility value set for the Patient Health Questionnaire depression scale in Hungary

**DOI:** 10.1192/j.eurpsy.2022.836

**Published:** 2022-09-01

**Authors:** P. Balázs, F. Rencz, V. Brodszky

**Affiliations:** Corvinus University of Budapest, Department Of Health Economics, Budapest, Hungary

**Keywords:** time trade-off, PHQ-9, health state measurement, value set

## Abstract

**Introduction:**

Depression is associated with high impact on health-related quality of life (HRQoL). Health state valuations are used for cost-effectiveness analysis to provide results for health-policy interventions.

**Objectives:**

The study aims to estimate a population-based value set of depression described by Patient Health Questionnaire (PHQ-9). We intend to assess vignettes describing PHQ-9 health states to estimate utility values

**Methods:**

Current research elicited direct utility scores using time trade-off (TTO) method obtained from the Hungarian general population (N=2,000). TTO vignettes were created to describe hypothetical health states of depression based on the nine items of PHQ-9. The hypothetical health states were sorted orthogonally in 11 blocks, each containing 4 vignettes (combinations of no; mild; moderate; severe depression). All respondents valued the four health states of one randomly given block. Conventional TTO method was applied, using a 10-year timeframe, while the first iteration step was randomized to 1,3 and 5 year. Preference weights were estimated using regression model fitted to TTO utility results.

**Results:**

Altogether 1,999 respondents valued overall 34 different health states. The mean age was 47.3 (16.9) years, the majority was female (57.2%). Nearly half of the respondents were secondary educated 45.4%, 27.3% higher educated and 27.2% completed primary school. The mean TTO utility of selected mild, moderate and severe depression was: 0.83; 0.82 and 0.77 respectively.

**Conclusions:**

Our results constitute the first population-based value set for PHQ-9. Utility scores give useful information for cost-effectiveness assessments. Estimates provide preference-based quality of life weights for the Hungarian population.

**Disclosure:**

No significant relationships.

